# Occupational Exposures and Risks of Non-Hodgkin Lymphoma: A Meta-Analysis

**DOI:** 10.3390/cancers15092600

**Published:** 2023-05-04

**Authors:** Luiza Flavia Veiga Francisco, Rogério Nunes da Silva, Marco Antônio Oliveira, Martins Fideles dos Santos Neto, Iara Zapparoli Gonçalves, Márcia M. C. Marques, Henrique C. S. Silveira

**Affiliations:** 1 Molecular Oncology Research Center, Barretos Cancer Hospital, Barretos 14784-390, SP, Brazil; luizaveiga10@hotmail.com (L.F.V.F.); u07073@hcancerbarretos.com.br (M.A.O.); martins_neto17@hotmail.com (M.F.d.S.N.); mmcmsilveira@gmail.com (M.M.C.M.); 2 Postgraduate Program in Environment and Health, University of Cuiabá, Cuiabá 78008-000, MT, Brazil; rogeryons@hotmail.com; 3 Hematology Department, Barretos Cancer Hospital, Barretos 14784-390, SP, Brazil; izap@uol.com.br; 4 Campus São Paulo, University of Anhanguera, São Paulo 04119-901, SP, Brazil

**Keywords:** non-Hodgkin lymphoma, occupational exposure, pesticides, carcinogens

## Abstract

**Simple Summary:**

It is unclear what causes the increased incidence of non-Hodgkin lymphoma (NHL); however, chemical substance exposure is known to be one of the risk factors for the disease. The aim of our systematic review was to verify the association between occupational exposure to carcinogens and NHL risk. In our literature review, 51 articles were included in the meta-analysis resulting in an overall OR of 1.27 (95% CI 1.04–1.55). Among these studies, 20 reported a significant association with the increased risk of NHL. We demonstrate that the risk of NHL increases for individuals occupationally exposed to pesticides, benzene, and trichloroethylene. Our findings may provide information for public health and practical decision-making about certain work activities and the use of chemical compounds.

**Abstract:**

Non-Hodgkin lymphoma (NHL) is a heterogeneous group with different types of diseases. It remains unclear as to what has led to an increase in incidences of NHL, however, chemical substance exposure is known to be one of the risk factors for the disease. Therefore, we performed a systematic review and meta-analysis including case-control, cohort, and cross-sectional observational epidemiological studies to verify the association between occupational exposure to carcinogens and NHL risk. Articles between the years 2000 and 2020 were collected. Two different reviewers performed a blind selection of the studies using the Rayyan QCRI web app. Post-completion, the selected articles were extracted and analyzed via the RedCap platform. Our review resulted in 2719 articles, of which 51 were included in the meta-analysis, resulting in an overall OR of 1.27 (95% CI 1.04–1.55). Furthermore, it was observed that the main occupation associated with the increased risk of NHL was that in which workers are exposed to pesticides. We therefore conclude that the evidence synthesis of the epidemiological literature supports an increased risk for NHL, regardless of subtype, considering occupational exposure to certain chemical compounds, mainly pesticides, benzene, and trichlorethylene, and certain classes of work, primarily in the field of agriculture.

## 1. Introduction

Non-Hodgkin lymphomas (NHL) are a heterogeneous group of diseases that stem from the proliferation of malignant lymphocytes and their precursors. These cells accumulate in the lymph nodes, but can also extend to other organs [1,2]. There are different subtypes of NHL, often with variations in their presentations, prognoses, and clinical treatments [3]. NHL ranks as the eighth and tenth most frequent cancer among men and women worldwide, respectively, with as many as 544,000 new cases and 260,000 deaths estimated for 2020 [4]. According to the data from Sung et al. [4] the highest incidence rates for NHL were found in Australia, New Zealand, North America, and Europe.

Etiologies of most NHL’s remain largely unknown, however, risk factors are likely to include immune deficiencies, Epstein-Barr virus, HTLV1, and Helicobacter pylori bacteria, radiation, chemicals, such as benzene, persistent organic pollutants, and pesticides [5,6]. Several environmental exposures have been suggested and investigated as factors that can potentially lead to the increased risk of NHL and play a crucial role in the increase of incidence of cases. 

Many recent studies have examined the association between environmental or occupational exposure to chemical compounds and the risk of developing NHL and specific subtypes [7,8,9,10,11]. In addition, many occupations have been investigated to establish the relationship between exposure and NHL risk [12,13,14,15,16,17,18]. Nonetheless, further studies are needed that evaluate the association between the increased risk of NHL and occupations in which chemical exposure is prevalent. 

There have been several review and meta-analysis studies focusing on the association between environmental pollutants and NHL [19,20,21,22], however, as of yet, no such review with a meta-analysis has been published featuring studies evaluating the association between a comprehensive number of work classes, chemical agents, and the risk of NHL. In short, the evidence regarding the link between occupations and chemical compounds and the development of NHL has not been completely understood to date. As such, we have conducted a systematic review, through meta-analysis, of observational epidemiological case-control, cohort, and cross-sectional studies to identify occupational classes of workers exposed to chemical agents associated with risks of NHL.

## 2. Materials and Methods

The systematic review and meta-analysis were performed according to the guidelines specified by PRISMA [23,24]. The review protocol was registered in the PROSPERO database (ID: CRD42020160291).

### 2.1. Search Strategy

Meta analysis was conducted to identify which types of workers were occupationally exposed to chemical agents that were potentially linked to the development of non-Hodgkin lymphomas. A systematic literature search of articles in the Medline, Cochrane Library, Virtual Health Library-BVS Regional Gateway, Scopus, PubMed, and Embase databases, from 2000 to 2020, was conducted. The search included the keywords “environmental carcinogens”, “iarc classification”, “occupation cancer”, “non-Hodgkin’s lymphoma”, “carcinogenic agents”, “environmental factors”, “occupational cancer”, “occupational exposure”, “workers”. Details of this search strategy are reported in Supplementary Material.

### 2.2. Study Selection and Data Extraction

Studies that fulfilled the specific inclusion criteria were included in the meta-analysis: (a) case-control, cross-sectional, prospective cohort, and retrospective type observational epidemiological studies; (b) original studies based on workers exposed to carcinogens; studies whose outcome was NHL; (c) articles written in English only; (d) studies in which the association between occupational exposure and the risk of NHL were expressed as relative risk (RR), standardized mortality ratios (SMR), hazard risk (HR), standardized incidence ratios (SIR), proportional mortality ratio (PMR), odds ratio (OR) with 95% confidence intervals (CIs), either reported or could be obtained from the data reported in the article. Studies such as meta-analysis, reviews, meetings, abstracts, letters, and comments were not included in this review. 

Study selection was performed independently by two reviewers using the blind system using the Rayyan QCRI online web app software [25] to categorize, include, and exclude eligible articles during the preliminary screening process based on the titles and abstracts. After the initial screening step, the entire text of the articles, which may contain relevant information, was reviewed by the two reviewers. The following information was extracted from the eligible articles: authorship, year of publication, country of publication and study, study design, population, gender of participants, diagnosis, occupational activity, chemical agent, sample size, number of exposed and non-exposed individuals (cohort and cross-sectional studies), number of case and control (case-control study), number of exposed individuals who developed NHL, and outcome measures. The RedCap web platform was used for data extraction by both reviewers and tabulated electronically. During each step, the results were compared, and any discrepancies were clarified with the participation of a third reviewer. 

### 2.3. Quality Assessment

Study quality assessment was carried out using the Study Quality Assessment Tool, developed by methodologists at the National Heart, Lung, and Blood Institute (NHLBI), in conjunction with professionals at the Research Triangle Institute International. The tool was based on other assessment tools created by the Evidence Based Practice Centers of the Agency for Healthcare Research and Quality (AHRQ), Cochrane, the Scottish Intercollegiate Guidelines Network and, others working in the context of evidence-based medicine. Designed to assist reviewers, the tool was considered, for this research, fundamental to the critical evaluation of the internal validity of a study. This includes items to assess potential flaws in study methods, including risks of bias.

During the quality review of the studies, each item of the tool may be answered with a “yes”, “no”, or “cannot be determined/not reported/not applicable”. For each item in which “no” was selected, a potential risk of bias was considered. This assessment tool is not designed to provide a list of factors that make up a numerical score or to delimit a score for reviewers. Thus, it is used, by following with the literature, for intervention studies, a rating of bad ≤ 6, regular > 6 and 5, and good ≥ 10 is used. For case-control studies that contained 12 questions instead of 14, the rating was adjusted: bad ≤ 5, regular > 5, and <9, and good ≥ 9 [26].

### 2.4. Data Analysis of the Systematic Review

Results from the cohort, cross-sectional, and case-control studies that associated exposure to chemical compounds or work class with increased risk of NHL were included in the meta-analysis. The meta-analysis was performed for each comparison using the random effects model, and the OR with the 95% Confidence Interval (CI) was calculated using the Mantel Haenszel method. The combined OR (${\hat{\psi }}_{MH}$) was estimated by adding together the individual ORs (${\hat{\psi }}_{k}$) from each study according to the expression of [27]:

(1) $${\hat{\psi }}_{MH}\;=\;\frac{\sum_{k=1}^{K}w_{k}{\hat{\psi }}_{k}}{\sum_{k=1}^{K}w_{k}}$$

where:

*w_k_* = (*b_k_ c_k_*)/*n_k_*
*K* = total number of studies
*k* = 1, 2,…, *K*
*ψ_k_* = (a_k_ d_k_)/(b_k_ c_k_)
*n_k_* = study sample size *k*
*a_k_* = number of events in the group exposed in the study *k*
*b_k_* = number of non-events in the group exposed in the study *k*
*c_k_* = number of events in the unexposed group in the study *k*
*d_k_* = number of non-events in the non-exposed group in the study *k*
*n_k_* = sample size (all studies)

Variables included in the meta-analysis were sample size, number of exposed individuals, number of non-exposed individuals, number of exposed individuals with an event, and non-exposed individuals with an event. Heterogeneity between each study was estimated using the Q-statistic of the Q-Cochran test and quantified using the inconsistency index (I^2^) [28]. Funnel plot was used to assess potential publication bias [29]. Analyses were performed using R version 4.1.2 (1 November 2021) software, using the functions metabin, forest.meta, and funnel from the package meta.

## 3. Results and Discussion

### 3.1. Characterization of the Studies

**Figure 1:**
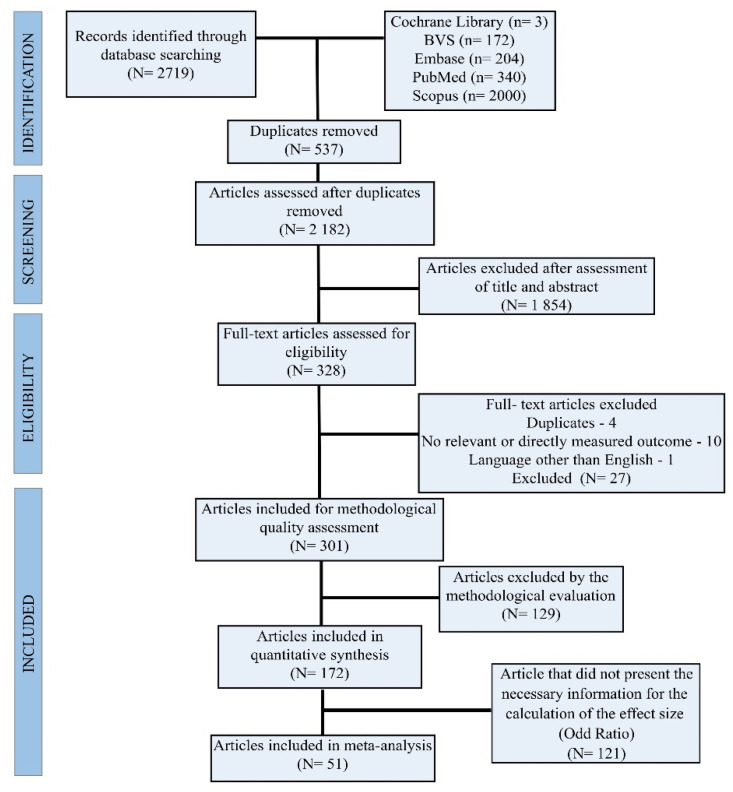
Flowchart of search and selection of articles included in the review and meta-analysis according to PRISMA.

The search of the database resulted in 2719 articles. Duplicate and irrelevant articles were excluded as described in the inclusion criteria. A total of 537 duplicate articles were excluded while the remaining 2182 were selected for evaluation based on titles and abstracts. Evaluation of the titles and abstracts revealed that 1854 articles did not analyze the association between occupations and carcinogens with the increased risk of NHL. As a result, an additional 1854 articles were excluded. The remaining 328 articles were subjected to a full-text review; of these, 27 articles did not meet the inclusion criteria. During data extraction, 129 articles were excluded by methodological evaluation, as a result, only 172 remained eligible for the study. Ultimately, 51 articles were included in the meta-analysis, because 121 articles did not present the necessary information for the calculation of the effect size (Odd Ratio) (Figure 1).

**Figure 2:**
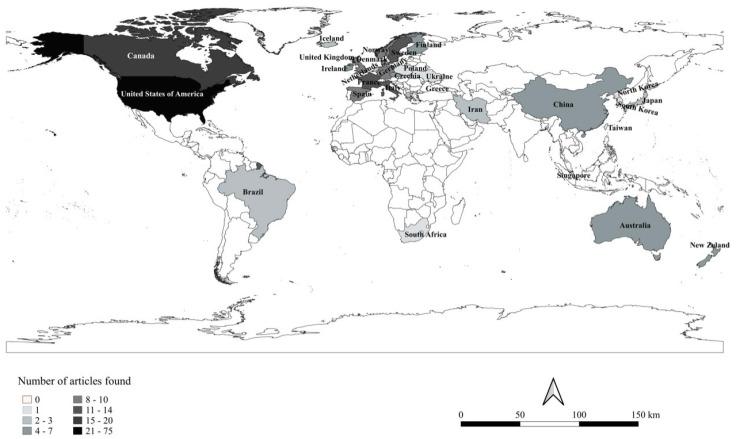
Location of studies included in the review that investigated the association of occupational exposure and NHL.

The systematic review comprised 76 case-control articles and 96 cohort/transversal articles. In regards to geographic coverage, of the 172 articles included in this review, 74 studies were conducted in the United States, 17 in Canada, 14 in France, 13 in Italy and Sweden, 10 in Germany and Spain, 9 in Denmark and Norway, 7 in China and the United Kingdom, 6 in Australia, New Zealand, and the Czech Republic, 5 in Finland and Ireland, 4 in Japan, 3 in Brazil, England, and Iceland, 2 in Korea, Greece, the Netherlands, and Iran, 1 in South Africa, Singapore, Poland, Taiwan, and Ukraine (Figure 2).

Several studies conducted at these locations are cohort studies or are part of consortia. The Agricultural Health Study (AHS) was the main cohort study evaluated in the studies [30,31,32,33,34,35,36,37,38,39,40,41,42]. The AHS is a prospective study of licensed pesticide applicators and their spouses in Iowa and North Carolina (USA), designed to understand how agricultural, lifestyle, and genetic factors affect the health of agricultural populations. The International Lymphoma Epidemiology Consortium (InterLymph) was found to have the largest number of studies among the consortia analyzed for this review [8,13,14,18,43,44,45]. The InterLymph Consortium conducts research through case-control studies in non-Hodgkin lymphoma addressing genetics, immunity and infection, lifestyle and environment, pathology, and survival. These types of studies have contributed greatly over the years to the understanding of the association between environmental/occupational exposure and the development of diseases such as non-Hodgkin lymphomas. 

The association between a large number of work classes and chemical compounds associated with the development of NHL and its subtypes has not been evaluated in any review. Reviews typically evaluate the association between the increased risk of overall NHL or some specific subtype with only one group of chemical agents or work class [21,22,46,47]. In our review, the following occupations: painter, driver, construction worker, hairdresser, chemical industry employee, solvent-exposed employees, agricultural mechanics and laborers, husbandry workers, and fishing laborers were the most investigated work classes used in the studies included in the review. In terms of the chemical compounds investigated in the studies, the most frequently evaluated were pesticides, radiation, solvents, hydrocarbons, metals, organic compounds, asbestos, paints, petroleum products, and organochlorines. 

### 3.2. Characteristics of the Studies Included in the Meta-Analysis

A total of 51 observational studies that met our inclusion criteria were identified and included in the meta-analysis. Accordingly, 28 case-control studies and 23 cohort studies were included. Table 1 shows an evaluated summary of the data extracted from each study, including author and year of publication, site, study design, exposure categories (work class and carcinogen), NHL subtype, and OR. Given the types of exposures present in the articles, the primary class of work evaluated in the articles, as part of the meta-analysis, was agricultural occupation and related activities. The main chemical agents analyzed were pesticides and solvents. When analyzing the NHL subtypes evaluated in the articles, of the 51 articles included in the meta-analysis, 22 evaluated the risk of Chronic Lymphocytic Leukemia/Small Cell Lymphocytic Lymphoma (CLL/SLL), 20 of Multiple Myeloma (MM), 17 of Follicular Lymphoma (FL) and Diffuse Large B Cell Lymphoma (DLBCL), and 6 of B cell in general. Although these were the most commonly investigated subtypes in the articles included in the meta-analysis, other subtypes of NHL were also evaluated as noted in Table 1.

**Figure 3:**
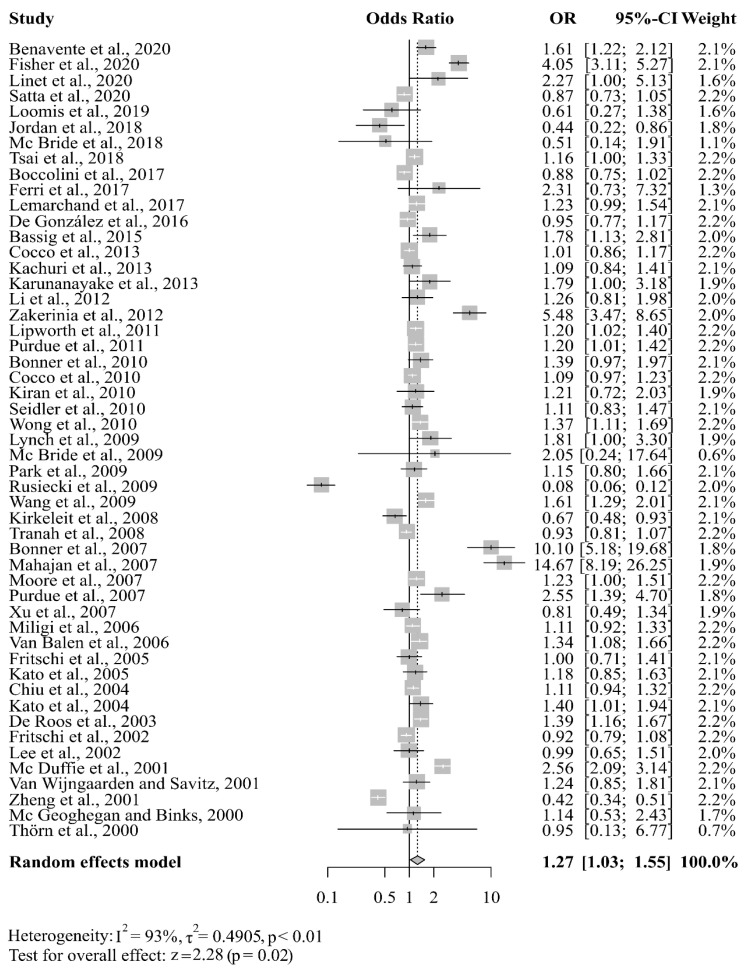
Forest plot of the overall odds ratio (OR) of random effects of occupational exposure and NHL risk [7,8,9,11,12,32,33,37,38,39,40,41,42,48,49,50,51,52,53,54,55,56,57,58,59,60,61,62,63,64,65,66,67,68,69,70,71,72,73,74,75,76,77,78,79,80,81,82,83,84,85].

The meta-analysis of the 51 studies produced an overall OR of 1.27 (95% CI 1.03–1.55). This suggests that some work classes and occupational exposure to certain compounds are associated with a 27% increase in the risk of NHL. The highest OR entered was for the study by Mahajan et al. [38] (OR = 14.67), in which they assessed exposure to pesticides. The lowest was for the study by Rusiecki et al. [41] (OR = 0.08), in which they assessed individuals in agricultural occupations. No individual study received more than 3% of the total weight assigned in the random effects model (Figure 3). The random effects model yielded a heterogeneity value of 93% with *p* < 0.01, indicating significant heterogeneity across studies (Figure 3 and Figure S1).

Altogether, more than 20 different work classes and more than 30 chemical compounds were evaluated (Table 1). Of the 20 studies that exhibited significant OR, most (14 studies) evaluated individuals in the agricultural occupation, or occupationally exposed to pesticides. As for the carcinogenic potential of the compounds most evaluated in the articles that were included in the meta-analysis, pesticides present varied carcinogenicity classifications, given that the classification depends greatly on the active compound evaluated. The only pesticide analyzed in the articles that were included in the meta-analysis that is classified as carcinogenic for humans (Group 1), according to the International Agency for Research on Cancer [86], is Lindane, which exhibited sufficient evidence of carcinogenicity for non-Hodgkin lymphoma [50].

Although pesticides constitute the main exposure evaluated in the articles, four of the 20 articles that revealed significant OR had evaluated exposure to solvents, formaldehyde, trichloroethylene, and benzene, which were the main compounds analyzed in these articles. In addition, significant OR was also observed for studies that evaluated employees in the manufacture and operation of aircraft, employees exposed to meat, and factory workers exposed to benzene (Table 1). Formaldehyde, trichloroethylene, and benzene solvents are considered carcinogenic to humans according to the IARC classification based on the results of epidemiological studies [86].

**Figure 4:**
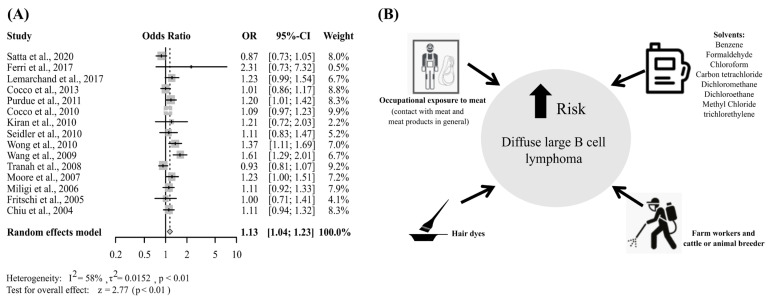
(**A**) Forest plot of the overall OR of occupational exposure and the risk of Diffuse Large B Cell Lymphoma; (**B**) Work classes and chemical compounds significantly associated with increased risk of Diffuse Large B Cell Lymphoma [8,9,49,55,56,63,64,65,66,68,70,71,73,74,76].

Given the high heterogeneity observed in our meta-analysis, we carried out subgroup analyses as an approach to identify the potential sources of heterogeneity in our overall meta-effects estimate. Thus, combined estimates of studies by NHL subtype, experimental design types, and exposure types were conducted. In the analysis performed for each NHL subtype evaluated in more than three studies, included in our meta-analysis, a meta-OR above 1.0 with significance for the random effects model was only observed for the DLBCL (OR 1.13, 95% CI 1.04–1.23) (Figure 4A and Figure S2).

The most frequent subtype of non-Hodgkin lymphoma worldwide is DLBCL which presents as a heterogeneous disease group with variable outcomes [87]. In our meta-analysis, the individual studies that showed a significantly increased risk of DLBCL (OR > 1), analyzed populations exposed to solvents (benzene, formaldehyde, chloroform, carbon tetrachloride, dichloromethane, dichloroethane, and methyl chloride) [64,70] and hair dyes [66]. In addition, one study carried out their investigations with meat handlers, people in contact with meat and meat products in general (contact with beef, chicken, pork, lamb, meat from other animals and fish), [73] and another study with the agricultural occupations (farm workers and cattle or animal breeders) [66] (Figure 4B). Few meta-analysis studies separately assess the DLBCL subtype associated with any given type of exposure. One of the compounds evaluated by the articles that showed significant OR in our meta-analysis for the DLBCL subtype was benzene. Rana et al. [88] found an association between benzene exposure and an increased risk of DLBCL in their meta-analysis.

The remaining meta-analyses for the remaining subtypes generated meta-OR above the null value of 1.0, yet, with no significance (Figure S3). Some meta-analysis studies assessed the increased risk of NHL subtypes associated with exposures to chemical compounds. Similar to our study, increased risks of MM (OR = 1.16; 95% CI = 0.99–1.36) were detected in association with pesticide exposure [89] and trichloroethylene [90], however, without statistical significance. MM risk was also not significant for occupational exposure to polycyclic aromatic hydrocarbons [91]. On the other hand, in the meta-analysis by Chang and Delzell [92], they found significant meta-relative risks for the association between glyphosate and MM, however, for the subtypes DLBCL, LLC/SLL, FL, and Hairy Cell Leukemia, no significant association was observed.

A significant association between exposure to any single solvent and the risk of FL was also identified [21]. In our study, the meta-analysis for FL showed positive association (OR > 1) however, of no significance (OR = 1.17, CI: 0.95–1.44). We did observe a significant association for individual studies, including two studies that evaluated solvent exposure [63,68] (Figure S3C). Many studies have also demonstrated an association between benzene exposure and subtypes of NHL. Benzene exposure has been associated with an increased incidence of Cutaneous T Cell Lymphoma [93], and CLL [9].

**Figure 5:**
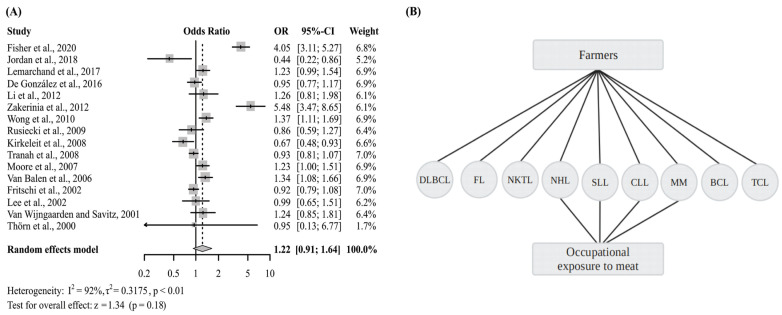
(**A**) Forest plot of the general OR of articles that only assessed work class and NHL risk; (**B**) Work classes significantly associated with increased risk of different subtypes of NHL. Diffuse Large B Cell Lymphoma (DLBCL), Follicular Lymphoma (FL), Natural killer/T-Cell Lymphoma (NKTL), Non-Hodgkin Lymphoma (NHL), Small Lymphocytic Lymphoma (SLL), Chronic Lymphocytic Leukemia (CLL), Multiple Myeloma (MM), B-cell Lymphomas (BCL), T-Cell Lymphoma (TCL) [11,12,41,42,51,56,57,61,66,69,70,71,79,80,82,85].

**Figure 6:**
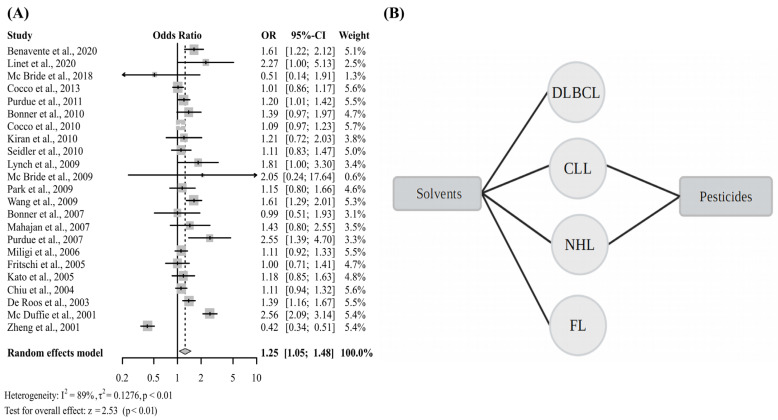
(**A**) Forest plot of the general OR of articles that evaluated only chemical compounds and the risk of NHL; (**B**) Chemical compounds significantly associated with increased risk of different subtypes of NHL. Diffuse large B Cell Lymphoma (DLBCL), Chronic Lymphocytic Leukemia (CLL), Non-Hodgkin Lymphoma (NHL), Follicular Lymphoma (FL) [7,8,9,32,33,37,38,39,40,48,52,63,64,65,67,68,73,74,75,76,78,81,83].

**Figure 7:**
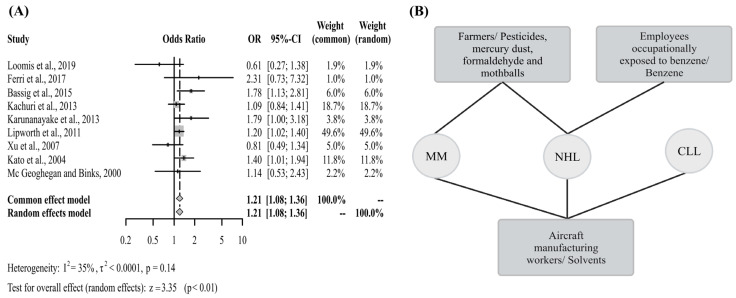
(**A**) Forest plot of the general OR of articles that evaluated work class and chemical compounds simultaneously and the risk of NHL; (**B**) Chemical compounds and work class significantly associated with increased risk of different NHL subtypes. Multiple Myeloma (MM), Non-Hodgkin Lymphoma (NHL), Chronic Lymphocytic Leukemia (CLL) [50,55,58,59,60,62,72,77,84].

When analyzing the different types of study designs, only the case-control studies analysis revealed a meta-OR above 1.0 with significance for the random-effects model (OR 1.21, 95% CI 1.03–1.41), while for the meta-analysis performed with the cohort studies, the meta-OR was less than 1.0 and non-significant (OR 0.82, 95% CI 0.36–1.92) (Figure S4). For the subgroup analyses by exposure type, three meta-analyses were performed, one considering studies that evaluated only work class, another evaluating only chemical compounds, and a third addressing only those articles that investigated both work class and exposure to chemical compounds (Figure 5, Figure 6 and Figure 7).

A meta-analysis, which included studies that analyzed only work class, did not exhibit significantly increased risk of NHL (OR 1.22, 95% CI 0.91–1.64) (Figure 5A and Figure S5). Despite not showing significant OR, five individual study results did show significant OR, among which, four assessed individuals in agricultural occupations [11,12,42,66] and one, of employees exposed to meat [71]. Furthermore, based on this analysis, NHL, B-Cell Lymphoma, DLBCL, FL, T-Cell, MM, CLL, SLL, and T/NK Cell, were the subtypes of NHL addressed by the studies that presented a significantly increased risk of NHL. These results illustrate the potential risks posed by these two classes of work concerning the development of NHL regardless of subtype, however, further studies are needed (Figure 5B).

For the meta-analysis that included studies that examined only chemical compounds, significantly increased risk of NHL was observed (OR 1.24, 95% CI 1.06–1.46) (Figure 6A and Figure S6). In this analysis, it was found that the studies with significant OR evaluated overall NHL, as well as the subtypes of DLBCL, FL, and CLL. Nine individual study results displayed significant OR, of which, five of the studies examined occupational exposure to pesticides [7,37,40,78,81]. A positive association between occupational exposure to some pesticides and the development of NHL was verified in the review study by Schinasi and Leon [94], in which they provided consistent evidence of this relationship. Exposure to glyphosate-based herbicides, 2,4-D and diazinon were also associated with increased risk of NHL in humans [22,46,47].

In addition to pesticide exposure, exposure to solvents, such as benzene (n = 1) [48], trichloroethylene (n = 1) [63], and solvents overall (n = 1) [68] (Figure 6B), was likewise observed in the studies. There is some evidence concerning the role of benzene in increasing the risk of NHL. In the meta-analysis studies by Rana et al. [88] and Steinmaus et al. [95], a causative link between benzene exposure and the development of NHL was evidenced. In contrast, the meta-analysis by Kane and Newton [96] found no association between benzene exposure and the increased risk of NHL or any subtype. Some studies have already demonstrated an association between exposure to trichloroethylene and the increased risk of NHL [63,93,97]. Our meta-analysis did not assess the individual association of compounds with the increased risk of NHL; however, we did find that these solvents were compounds evaluated in the studies that displayed a significant OR in our meta-analysis.

The meta-analysis that included studies that examined both work class and chemical compounds did exhibit a significantly increased risk of NHL (OR 1.21, 95% CI 1.08–1.36) (Figure 7A and Figure S7). The studies that showed significant OR in this analysis evaluated only the development of general NHL and/or MM and CLL subtypes. As for the exposure assessed in each of these studies, one of the studies looked at factory employees exposed to benzene along with the compound benzene [58]; another study evaluated individuals from the agricultural occupation, in addition to, pesticides, mercury, and formaldehyde [60]; there was another study with employees employed in aircraft manufacturing with exposure to chromate compounds, trichloroethylene, perchloroethylene, and mixed solvents [62]; and a further study included agricultural workers, and assessed exposure to pesticides and naphthalene [77] (Figure 7B).

Subgroup analyses such as NHL subtype, study design, and exposure type did not reveal the leading sources of heterogeneity observed in our overall meta-analysis (Figure 3 and Figure S1). Nevertheless, considering the variety of NHL subtypes, the different possible settings for exposure (work class and chemical agents), exposure assessment methods, statistical results, study population, study site, and other factors evaluated in the studies included in our meta-analysis, we had already anticipated a high heterogeneity. In summary, despite the heterogeneity between studies in the different analyses performed and in our overall meta-analysis (Figure S1), we conclude that the synthesis of evidence from the epidemiological literature supports an increased risk for NHL. This result is independent of the subtype of NHL and the type of occupational exposure and compounds evaluated.

### 3.3. Strengths

Our meta-analysis synthesized 51 epidemiological studies examining the relationship between chemical agents and work classes and the risk of NHL. To the best of our knowledge, this is the first and largest systematic review with meta-analysis that takes into account all exposure types (chemical agents and/or work class) and all subtypes of NHL. Overall, our results provide evidence for the hypothesis that occupational exposure to chemical agents increases the risk of NHL. Thus, these results represent an important contribution to the literature on exposures associated with the development of NHL.

### 3.4. Limitations

A limitation of the study is that our review was conducted with studies published between 2000 and 2020 (which included mature B-, T-, and NK-cell neoplasms as well as CLL and multiple myeloma as subtypes of LNH). However, according to the new classification of the World Health Organization to Hematolymphoid Tumors: Lymphoid Neoplasms (5th edition), the term LNH is no longer used [98]. The 51 studies that were reviewed in the analysis were quite heterogeneous, leading to high heterogeneity in the overall and subgroup meta-analysis. Although we examined possible sources of heterogeneity using factors such as NHL subtype, exposure type, and study design, there may be other possible causes of heterogeneity that could not be assessed. Adjustment for confounding factors such as anthropometric and sociodemographic variables was not performed, as most of the studies included had adjusted their risk measure for such factors. It is important to note that people are subject to both occupational and environmental exposure to a mixture of chemical compounds, causing the exposure profile to not be fully characterized in individual studies, and thus some of the observed associations may be due to chance. A further limitation of our study is that exposure, in most of the studies, was assessed by self-reported responses to epidemiologically applied questionnaires, which have the potential for memory bias and measurement error and not by objective and standardized measurements for exposure.

## 4. Conclusions

In this systematic review through meta-analysis, we present the evidence, through a detailed evaluation of epidemiologic studies, supporting the association between occupational chemical exposure and the risk of developing non-Hodgkin lymphoma. We demonstrate that the risk of NHL, regardless of the subtype, increases for individuals occupationally exposed to chemical agents, mainly pesticides, benzene, and trichloroethylene, as well as for certain work classes, primarily for occupations in agriculture. However, there is still insufficient data on the association between NHL and specific chemical compounds. Our findings may provide information for public health and practical decision-making about certain work activities and the use of chemical compounds. Furthermore, the evidence for the association of specific chemical compound classes and work classes associated with the development of NHL in biological samples is still limited, so future mechanistic studies, measuring exposures, and evaluating the biological and molecular effects associated with the risk of NHL are still needed.

## Figures and Tables

**Table 1:** Characteristics of the studies included in the meta-analysis.

Authors	Country	Study Design	Work Class	Carcinogen	NHL Subtype	Odds Ration IC 95%
Benavente et al. (2020) [7]	Spain	Case-control	-	Pesticides	NHL; CLL	1.61 (1.22–2.12)
Fisher et al. (2020) [42]	USA	Cohort	- Pesticide Applicators	-	NHL; BC-NHL; CLL; SLL; DLBCL; FL; MM	4.05 (3.11–5.27)
Linet et al. (2020) [48]	China	Cohort		Benzene	NHL	2.27 (1.00–5.13)
Satta et al. (2020) [49]	Czech Republic, France, Germany, Ireland, Italy and Spain	Case-control	-	Internal and External Ionizing Radiation	BC-NHL; CLL; DLBCL; FL; MM	0.87 (0.73–1.05)
Loomis et al. (2019) [50]	Denmark, Finland, Italy, Norway, Sweden, and the United Kingdom	Cohort	- Workers in the reinforced plastic industry - Laminators	Styrene	NHL; MM	0.61 (0.27–1.38)
Jordan et al. (2018) [51]	USA	Cohort	- Search/Rescue Workers	-	NHL	0.44 (0.22–0.86)
McBride et al. (2018) [52]	New Zealand	Cohort	-	2,3,7,8-tetraclorodibenzo-*p*-dioxin (TCDD)	NHL; MM	0.51 (0.14–1.91)
Tsai et al. (2018) [53]	Taiwan	Cohort	- Farmers	-	NHL	1.16 (1.0–1.33)
Boccolini et al. (2017) [54]	Brazil	Case-control	- Farm Workers	-	NHL	0.88 (0.75–1.02)
Ferri et al. (2017) [55]	Italy	Case-control	- Homemaker - Blue-collar worker - Teachers - Craftsman/Merchant - Farmer - Clerk - Military - Technician - Food handlers - Agricultural occupation	Pesticides and radon	NHL; DLBCL; FL; CLL; Single B-cell Lymphoma; MM	2.31 (0.73–7.32)
Lemarchand et al. (2017) [56]	France	Cohort	- Farm Workers		NHL; CLL/SLL; FL; DLBCL; MCL; MZL; Waldentröm Lymphoplasmacytic Lymphoma; NK/T-CL; MF; NHL-NOS	1.23 (0.99–1.54)
González et al. (2016) [57]	USA	Cohort	- Radiologist		NHL; CLL; MM	0.95 (0.77–1.17)
Bassig et al. (2015) [58]	China	Cohort	- Factory Employees Exposed to Benzene	Benzene	NHL	1.78 (1.13–2.81)
Cocco et al. (2013) [8]	USA, Czech Republic, France, Germany, Italy, Ireland, Spain and Canada	Case-control	-	Trichloroethylene	NHL; DLBCL; FL; CLL	1.01 (0.86–1.17)
Kachuri et al. (2013) [59]	Canada	Case-control	- Farm workers	Pesticides and formaldehyde	MM	1.09 (0.84–1.41)
Karunanayake et al. (2013) [60]	Canada	Case-control	- Workers exposed to Pesticides	-	NHL	1.79 (1.0–3.18)
Li et al. (2012) [61]	USA	Cohort	- Search/Rescue Workers		NHL; MM	1.26 (0.81–1.98)
Zakerinia et al. (2012) [11]	Iran	Case-control	- Workers exposed to Pesticides	-	NHL; MM	5.48 (3.47–8.65)
Lipworth et al. (2011) [62]	USA	Cohort	- Employees of aircraft manufacturing - Aircraft painter - Process or electroplating operator - Plastic parts manufacturer - Welder - Metal bonding worker - Fabrication and structural development mechanic - Final Assembler	Chromate and solvents	NHL; MM; CLL	1.20 (1.02–1.40)
Purdue et al. (2011) [63]	USA	Case-control	-	Trichloroethylene	NHL; DLBCL; FL; SLL; CLL	1.20 (1.01–1.42)
Bonner et al. (2010) [32]	USA	Cohort	-	Terbufos	NHL	1.39 (0.97–1.97)
Cocco et al. (2010) [9]	Czech Republic, France, Germany, Ireland, Italy, and Spain	Case-control	-	Solvents	DLBCL; FL; CLL; MM; BC- NHL	1.09 (0.97–1.23)
Kiran et al. (2010) [64]	Czech Republic, France, Germany, Italy, Ireland, and Spain	Case-control	-	Ethylene	DLBCL; CLL	1.21 (0.72–2.03)
Seidler et al. (2010) [65]	Germany, and Italy	Case-control	-	Asbestos	BC-NHL; DLBCL; FL; CLL; MM	1.11 (0.83–1.47)
Wong et al. (2010) [66]	China e USA	Case-control	- Farmer - Livestock or Animal Husbandry	-	NHL; CLL; FL; DLBCL; T/NK Cell Neoplasms	1.37 (1.11–1.69)
Lynch et al. (2009) [37]	USA	Cohort	-	Butylate	NHL	1.81 (1.0–3.30)
McBride et al. (2009) [67]	New Zealand	Cohort	-	2,3,7,8-Tetrachlorodibenzo-*p*-dioxin	NHL; MM	2.05 (0.24–17.64)
Park et al. (2009) [39]	USA	Cohort	-	Paraquat	NHL	1.15 (0.80–1.66)
Rusiecki et al. (2009) [41]	USA	Cohort	- Farmers	-	NHL; MM	0.08 (0.06–0.12)
Wang et al. (2009) [68]	USA	Case-control	-	Solvent	NHL; DLBCL; FL; CLL	1.61 (1.29–2.01)
Kirkeleit et al. (2008) [69]	Norway	Cohort	- Oil industry workers	-	NHL; CLL; MM	0.67 (0.48–0.93)
Tranah et al. (2008) [70]	USA	Case-control	- Animal Husbandry - Farm Laborer	-	NHL; DLBCL or Immunoblastic Large Cell Lymphoma; FL; SLL	0.93 (0.81–1.07)
Bonner et al. (2007) [33]	USA	Cohort	-	Malathion	NHL	10.10 (5.18–19.68)
Mahajan et al. (2007) [38]	USA	Cohort	-	Carbaryl	NHL	14.67 (8.19–26.25)
Moore et al. (2007) [71]	Czech Republic, France, Germany, Ireland, Italy, and Spain	Case-control	- Meat handlers	-	NHL; MM; CLL; SLL; DLBCL; FL	1.23 (1.00–1.51)
Purdue et al. (2007) [40]	USA	Cohort	-	Pesticides	NHL	2.55 (1.39–4.70)
Xu et al. (2007) [72]	Japan, Korea and China	Case-control	- Farmers - Chemical Plant Workers - Self-Employed	Pesticides	Nasal T/NK-cell Lymphoma	0.81 (0.49–1.34)
Miligi et al. (2006) [73]	Italy	Case-control	-	Solvents and hydrocarbons	NHL; SLL; FL; DLBCL	1.11 (0.92–1.33)
Balen et al. (2006) [12]	Spain	Case-control	- Farmer - Animal Husbandry	-	NHL; B-Cell NHL; TC-NHL; MM	1.34 (1.08–1.66)
Fritschi et al. (2005) [74]	Australia	Case-control	-	Pesticides	NHL; BC- NHL; DLBCL; FL	1.00 (0.71–1.41)
Kato et al. (2005) [75]	USA	Case-control	-	Solvents	NHL	1.18 (0.85–1.63)
Chiu et al. (2004) [76]	USA	Case-control	-	Pesticides	NHL; DCL; SLL; FL	1.11 (0.94–1.32)
Kato et al. (2004) [77]	USA	Case-control	- Farm Workers - Pesticide Applicators	Pesticides and naphthalene	NHL	1.40 (1.01–1.94)
De Roos et al. (2003) [78]	USA	Case-control	-	Pesticides	NHL	1.39 (1.16–1.67)
Fritschi et al. (2002) [79]	Canada	Case-control	- Workers in contact with animals - Farm Workers - Fishermen	-	NHL; MM	0.92 (0.79–1.08)
Lee et al. (2002) [80]	USA	Cohort	- Farmers - Cattle Ranchers	-	NHL; MM; CLL	0.99 (0.65–1.51)
McDuffie et al. (2001) [81]	Canada	Case-control	-	Pesticides	NHL	2.56 (2.09–3.14)
VanWijngaarden and Savitz (2001) [82]	USA	Case-control	- Electric Utility Workers - Electric Utility Workers Exposed to Solvents	-	NHL; Low and high-grade NHL	1.24 (0.85–1.81)
Zheng et al. (2001) [83]	USA	Case-control	-	Pesticides	NHL; FL; Diffuse NHL; SLL	0.42 (0.34–0.51)
McGeoghegan and Binks (2000) [84]	England	Cohort	- Uranium Production and Manufacturing Workers	Radiation	NHL; MM	1.14 (0.53–2.43)
Thörn et al. (2000) [85]	Sweden	Cohort	- Lumberjack	-	NHL	0.95 (0.13–6.77)

NHL: Non-Hodgkin’s Lymphoma; CLL: Chronic Lymphocytic Leukemia; BC-NHL: B-Cell Non-Hodgkin’s Lymphoma; SLL: Small Cell Lymphocytic Lymphoma; DLBCL: Diffuse Large B Cell Lymphoma; FL: Follicular Lymphoma; MM: Multiple Myeloma; MCL: Mantle Cell Lymphoma; MZL: Marginal Zone Lymphoma; NK/T-CL: NK/T-cell Lymphoma; MF: Mycosis Fungoides; TC-NHL: T-Cell Non-Hodgkin’s Lymphoma; DCL: Diffuse Cell Lymphoma; USA: United States of America.

## Data Availability

Data will be made available on request.

## Supplementary Materials

The following supporting information can be downloaded at: https://www.mdpi.com/article/10.3390/cancers15092600/s1, Figure S1: Funnel Plot of the studies included in the overall meta-analysis. Each dot represents one study; Figure S2: Funnel Plot of the studies included in the meta-analysis for the Diffuse Large B Cell Lymphoma subtype. Each dot represents one study; Figure S3: Forest Plot and Funnel Plot of the studies included in the meta-analysis for (A) Multiple Myeloma subtype, (B) Chronic Lymphocytic Leukemia, (C) Follicular Lymphoma, and (D) B-Cell Non-Hodgkin’s Lymphoma; Figure S4: Forest Plot and Funnel Plot of the (A) Case-control, and (B) Cohort and cross-sectional studies included in the meta-analysis; Figure S5: Funnel Plot of the studies included in the meta-analysis that evaluated only work class. Each point represents one study; Figure S6: Funnel Plot of the studies included in the meta-analysis that evaluated only chemical compounds. Each point represents one study; Figure S7: Funnel Plot of the studies included in the meta-analysis that simultaneously evaluated work class and exposure to chemical compounds. Each dot represents one study.
