# Supplementary material for: Occupational Exposures and Risks of Non-Hodgkin Lymphoma: A Meta-Analysis
Source: Cancers (Basel). 2023 May 4;15(9):2600. doi: 10.3390/cancers15092600 (PMC10177442; doi:10.3390/cancers15092600)
Supplement: Supplementary file 1 [file cancers-15-02600-s001.zip › cancers-2300971-supplementary.pdf]

SUPPLEMENTARY MATERIAL

# Occupational Exposures and Risks of Non-Hodgkin Lymphoma: A Meta-Analysis

## Methods

### Search Strategy

("Lymphoma, Non-Hodgkin" OR "Lymphoma, Non Hodgkin" OR "Lymphoma, Small Cleaved-Cell, Diffuse" OR "Lymphoma, Atypical Diffuse Small Lymphoid" OR "Diffuse Small Cleaved-Cell Lymphoma" OR "Diffuse Small Cleaved Cell Lymphoma" OR "Lymphoma, Small Cleaved Cell, Diffuse" OR "Lymphoma, Nonhodgkins" OR "Nonhodgkins Lymphoma" OR "Lymphoma, Non-Hodgkins" OR "Lymphoma, Non Hodgkins" OR "Non-Hodgkins Lymphoma" OR "Non-Hodgkin's Lymphoma" OR "Non Hodgkin's Lymphoma" OR "Non-Hodgkin Lymphoma" OR "Non Hodgkin Lymphoma" OR "Lymphoma, Nonhodgkin's" OR "Lymphoma, Nonhodgkin" OR "Nonhodgkin's Lymphoma" OR "Small Cleaved-Cell Lymphoma, Diffuse" OR "Small Cleaved Cell Lymphoma, Diffuse" OR "Lymphoma, Non-Hodgkin's" OR "Lymphoma, Non Hodgkin's" OR "Lymphoma, Diffuse" OR "Diffuse Lymphoma" OR "Diffuse Lymphomas" OR "Sarcoma, Lymphatic" OR Lymphosarcoma OR Lymphosarcomas OR "Lymphatic Sarcoma" OR "Lymphatic Sarcomas" OR "Lymphoma, Low-Grade" OR "Low-Grade Lymphoma" OR "Low-Grade Lymphomas" OR "Lymphoma, Low Grade" OR "Lymphoma, Mixed-Cell" OR "Lymphoma, Mixed Cell" OR "Mixed-Cell Lymphoma" OR "Mixed Cell Lymphoma" OR "Mixed-Cell Lymphomas" OR "Lymphoma, Mixed" OR "Mixed Lymphoma" OR "Mixed Lymphomas" OR "Lymphoma, Mixed Lymphocytic-Histiocytic" OR "Lymphocytic-Histiocytic Lymphoma, Mixed" OR "Lymphocytic-Histiocytic Lymphomas, Mixed" OR "Lymphoma, Mixed Lymphocytic Histiocytic" OR "Mixed Lymphocytic-Histiocytic Lymphoma" OR "Mixed Lymphocytic-Histiocytic Lymphomas" OR "Lymphoma, Non-Hodgkin, Familial" OR "Lymphoma, Small Noncleaved-Cell" OR "Lymphoma, Small

Noncleaved Cell" OR "Small Noncleaved-Cell Lymphoma" OR "Noncleaved-Cell Lymphoma, Small" OR "Small Noncleaved Cell Lymphoma" OR "Small Noncleaved-Cell Lymphomas" OR "Diffuse Undifferentiated Lymphoma" OR "Diffuse Undifferentiated Lymphomas" OR "Lymphoma, Diffuse Undifferentiated" OR "Undifferentiated Lymphoma, Diffuse" OR "Lymphoma, Small Non-Cleaved-Cell" OR "Lymphoma, Small Non Cleaved Cell" OR "Non-Cleaved-Cell Lymphoma, Small" OR "Small Non-Cleaved-Cell Lymphomas" OR "Small Non-Cleaved-Cell Lymphoma" OR "Small Non Cleaved Cell Lymphoma" OR "Lymphoma, Undifferentiated, Diffuse" OR "Lymphoma, Intermediate-Grade" OR "Intermediate-Grade Lymphoma" OR "Intermediate-Grade Lymphomas" OR "Lymphoma, Intermediate Grade" OR "Lymphoma, Undifferentiated" OR "Lymphoma, Pleomorphic" OR "Pleomorphic Lymphoma" OR "Pleomorphic Lymphomas" OR "Undifferentiated Lymphoma" OR "Undifferentiated Lymphomas" OR "Reticulum-Cell Sarcoma" OR "Reticulum-Cell Sarcomas" OR "Sarcoma, Reticulum-Cell" OR "Sarcoma, Reticulum Cell" OR "Reticulum Cell Sarcoma" OR "Reticulum Cell Sarcomas" OR Reticulosarcoma OR Reticulosarcomas OR "Lymphoma, High-Grade" OR "High-Grade Lymphoma" OR "High-Grade Lymphomas" OR "Lymphoma, High Grade" OR "Lymphoma, Mixed-Cell, Diffuse" OR "Mixed Small and Large Cell Lymphoma, Diffuse" OR "Lymphoma, Mixed Small and Large Cell, Diffuse" OR "Lymphoma, Diffuse, Mixed Lymphocytic-Histiocytic" OR "Lymphoma, Small and Large Cleaved-Cell, Diffuse" OR "Lymphoma, Mixed Cell, Diffuse" OR "Mixed-Cell Lymphoma, Diffuse" OR "Mixed Cell Lymphoma, Diffuse" OR "Diffuse Mixed Small and Large Cell Lymphoma" OR "Diffuse Mixed-Cell Lymphoma" OR "Diffuse Mixed Cell Lymphoma" OR "Diffuse Mixed-Cell Lymphomas" OR "Lymphoma, Diffuse Mixed-Cell") AND ("Carcinogenic Agents" OR "Agents, carcinogenic" OR "Carcinogenic Drugs" OR "Drugs, Carcinogenic" OR carcinogens OR "Agents, Carcinogenic" OR "Carcinogenic Effect" OR "Effect, carcinogenic" OR Carcinogenesis OR Carcinogeneses OR Tumorigenesis OR Tumorigeneses OR Oncogenesis OR Oncogeneses) AND (Workers OR Worker)

## Results

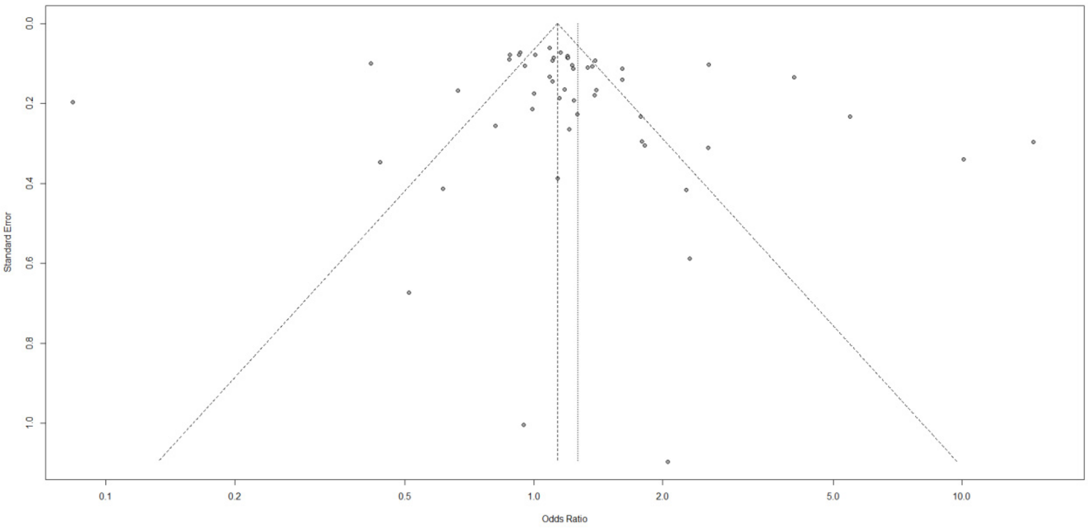


**Figure S1:** Funnel Plot of the studies included in the overall meta-analysis. Each dot represents one study.

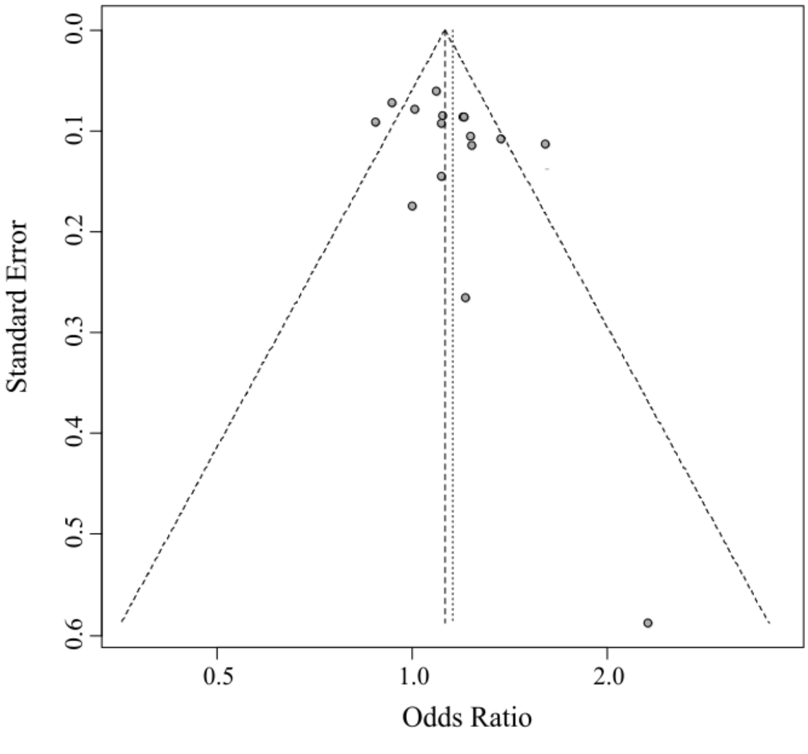


**Figure S2:** Funnel Plot of the studies included in the meta-analysis for the Diffuse Large B Cell Lymphoma subtype. Each dot represents one study.

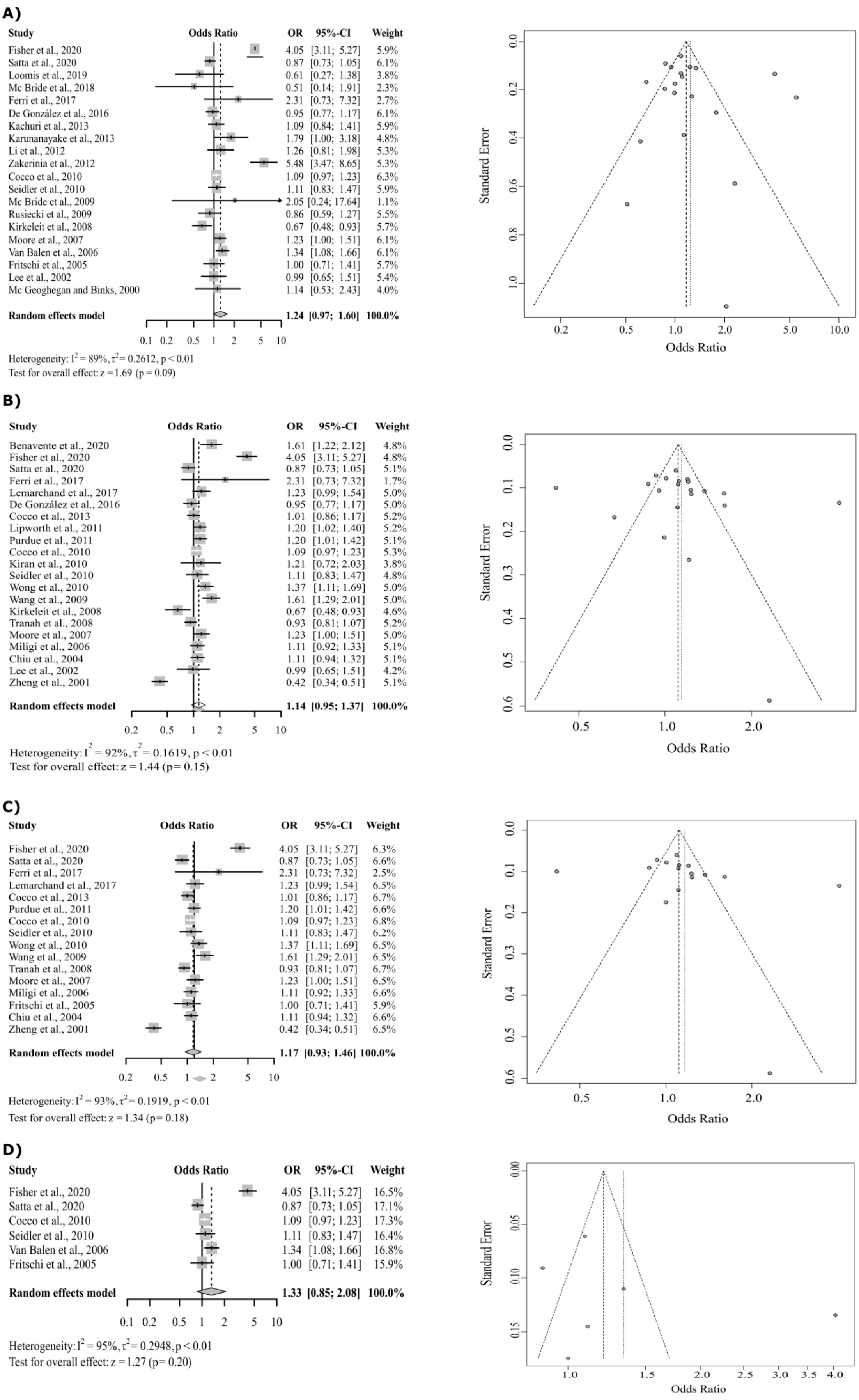
A)
Study
Odds Ratio
OR
95%-CI
Weight
Fisher et al., 2020 4.05 [3.11; 5.27] 5.9%
Satta et al., 2020 0.87 [0.73; 1.05] 6.1%
Loomis et al., 2019 0.61 [0.27; 1.38] 3.8%
Mc Bride et al., 2018 0.51 [0.14; 1.91] 2.3%
Ferri et al., 2017 2.31 [0.73; 7.32] 2.7%
De González et al., 2016 0.95 [0.77; 1.17] 6.1%
Kachuri et al., 2013 1.09 [0.84; 1.41] 5.9%
Karunanayake et al., 2013 1.79 [1.00; 3.18] 4.8%
Li et al., 2012 1.26 [0.81; 1.98] 5.3%
Zakerinia et al., 2012 5.48 [3.47; 8.65] 5.3%
Cocco et al., 2010 1.09 [0.97; 1.23] 6.3%
Seidler et al., 2010 1.11 [0.83; 1.47] 5.9%
Mc Bride et al., 2009 2.05 [0.24; 17.64] 1.1%
Rusiecki et al., 2009 0.86 [0.59; 1.27] 5.5%
Kirkeleit et al., 2008 0.67 [0.48; 0.93] 5.7%
Moore et al., 2007 1.23 [1.00; 1.51] 6.1%
Van Balen et al., 2006 1.34 [1.08; 1.66] 6.1%
Fritschi et al., 2005 1.00 [0.71; 1.41] 5.7%
Lee et al., 2002 0.99 [0.65; 1.51] 5.4%
Mc Geoghegan and Binks, 2000 1.14 [0.53; 2.43] 4.0%
Random effects model 1.24 [0.97; 1.60] 100.0%
0.1 0.2 0.5 1 2 5 10
Heterogeneity: $I^2$ = 89%, $\tau^2$ = 0.2612, p < 0.01
Test for overall effect: z = 1.69 (p = 0.09)
Standard Error
0.0 0.2 0.4 0.6 0.8 1.0
0.2 0.5 1.0 2.0 5.0 10.0
Odds Ratio
B)
Study
Odds Ratio
OR
95%-CI
Weight
Benavente et al., 2020 1.61 [1.22; 2.12] 4.8%
Fisher et al., 2020 4.05 [3.11; 5.27] 4.8%
Satta et al., 2020 0.87 [0.73; 1.05] 5.1%
Ferri et al., 2017 2.31 [0.73; 7.32] 1.7%
Lemarchand et al., 2017 1.23 [0.99; 1.54] 5.0%
De González et al., 2016 0.95 [0.77; 1.17] 5.0%
Cocco et al., 2013 1.01 [0.86; 1.17] 5.2%
Lipworth et al., 2011 1.20 [1.02; 1.40] 5.2%
Purdue et al., 2011 1.20 [1.01; 1.42] 5.1%
Cocco et al., 2010 1.09 [0.97; 1.23] 5.3%
Kiran et al., 2010 1.21 [0.72; 2.03] 3.8%
Seidler et al., 2010 1.11 [0.83; 1.47] 4.8%
Wong et al., 2010 1.37 [1.11; 1.69] 5.0%
Wang et al., 2009 1.61 [1.29; 2.01] 5.0%
Kirkeleit et al., 2008 0.67 [0.48; 0.93] 4.6%
Tranah et al., 2008 0.93 [0.81; 1.07] 5.2%
Moore et al., 2007 1.23 [1.00; 1.51] 5.0%
Miligi et al., 2006 1.11 [0.92; 1.33] 5.1%
Chiu et al., 2004 1.11 [0.94; 1.32] 5.1%
Lee et al., 2002 0.99 [0.65; 1.51] 4.2%
Zheng et al., 2001 0.42 [0.34; 0.51] 5.1%
Random effects model 1.14 [0.95; 1.37] 100.0%
0.2 0.5 1 2 5 10
Heterogeneity: $I^2$ = 92%, $\tau^2$ = 0.1619, p < 0.01
Test for overall effect: z = 1.44 (p= 0.15)
Standard Error
0.0 0.1 0.2 0.3 0.4 0.5 0.6
0.5 1.0 2.0
Odds Ratio
C)
Study
Odds Ratio
OR
95%-CI
Weight
Fisher et al., 2020 4.05 [3.11; 5.27] 6.3%
Satta et al., 2020 0.87 [0.73; 1.05] 6.6%
Ferri et al., 2017 2.31 [0.73; 7.32] 2.5%
Lemarchand et al., 2017 1.23 [0.99; 1.54] 6.5%
Cocco et al., 2013 1.01 [0.86; 1.17] 6.7%
Purdue et al., 2011 1.20 [1.01; 1.42] 6.6%
Cocco et al., 2010 1.09 [0.97; 1.23] 6.8%
Seidler et al., 2010 1.11 [0.83; 1.47] 6.2%
Wong et al., 2010 1.37 [1.11; 1.69] 6.5%
Wang et al., 2009 1.61 [1.29; 2.01] 6.5%
Tranah et al., 2008 0.93 [0.81; 1.07] 6.7%
Moore et al., 2007 1.23 [1.00; 1.51] 6.5%
Miligi et al., 2006 1.11 [0.92; 1.33] 6.6%
Fritschi et al., 2005 1.00 [0.71; 1.41] 5.9%
Chiu et al., 2004 1.11 [0.94; 1.32] 6.6%
Zheng et al., 2001 0.42 [0.34; 0.51] 6.5%
Random effects model 1.17 [0.93; 1.46] 100.0%
0.2 0.5 1 2 5 10
Heterogeneity: $I^2$ = 93%, $\tau^2$ = 0.1919, p < 0.01
Test for overall effect: z = 1.34 (p= 0.18)
Standard Error
0.0 0.1 0.2 0.3 0.4 0.5 0.6
0.5 1.0 2.0
Odds Ratio
D)
Study
Odds Ratio
OR
95%-CI
Weight
Fisher et al., 2020 4.05 [3.11; 5.27] 16.5%
Satta et al., 2020 0.87 [0.73; 1.05] 17.1%
Cocco et al., 2010 1.09 [0.97; 1.23] 17.3%
Seidler et al., 2010 1.11 [0.83; 1.47] 16.4%
Van Balen et al., 2006 1.34 [1.08; 1.66] 16.8%
Fritschi et al., 2005 1.00 [0.71; 1.41] 15.9%
Random effects model 1.33 [0.85; 2.08] 100.0%
0.1 0.2 0.5 1 2 5 10
Heterogeneity: $I^2$ = 95%, $\tau^2$ = 0.2948, p < 0.01
Test for overall effect: z = 1.27 (p= 0.20)
Standard Error
0.00 0.05 0.10 0.15
1.0 1.5 2.0 2.5 3.0 3.5 4.0
Odds Ratio

**Figure S3:** Forest Plot and Funnel Plot of the studies included in the meta-analysis for (A) Multiple Myeloma subtype [1–20]; (B) Chronic Lymphocytic Leukemia [1,2,5,6,11–13,16,21–33]; (C) Follicular Lymphoma [1,2,5,11–13,18,22,23,25,27–31,33]; and (D) B-Cell Non-Hodgkin's Lymphoma [1,2,12,13,17,18].

**A)**

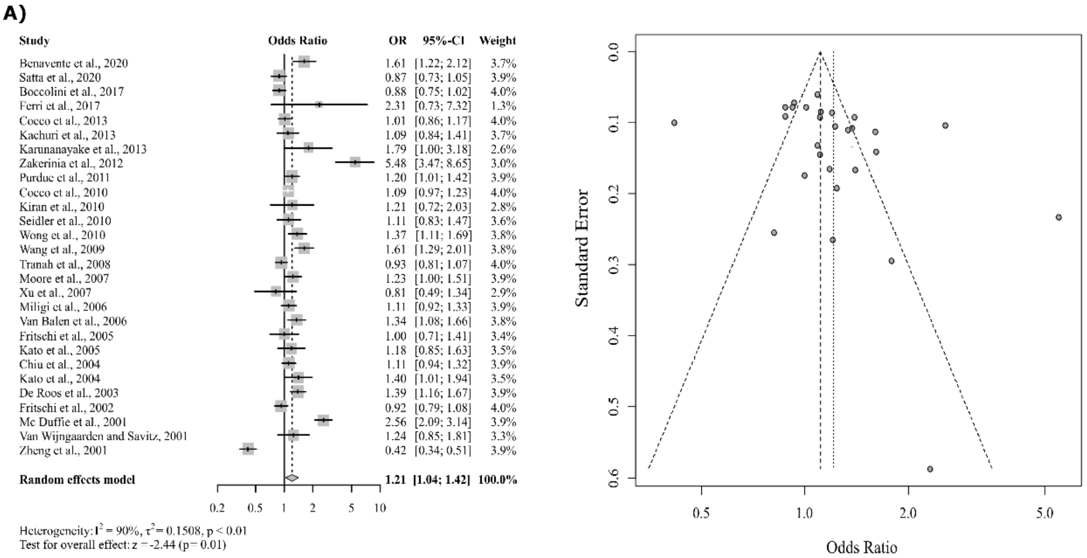


**B)**

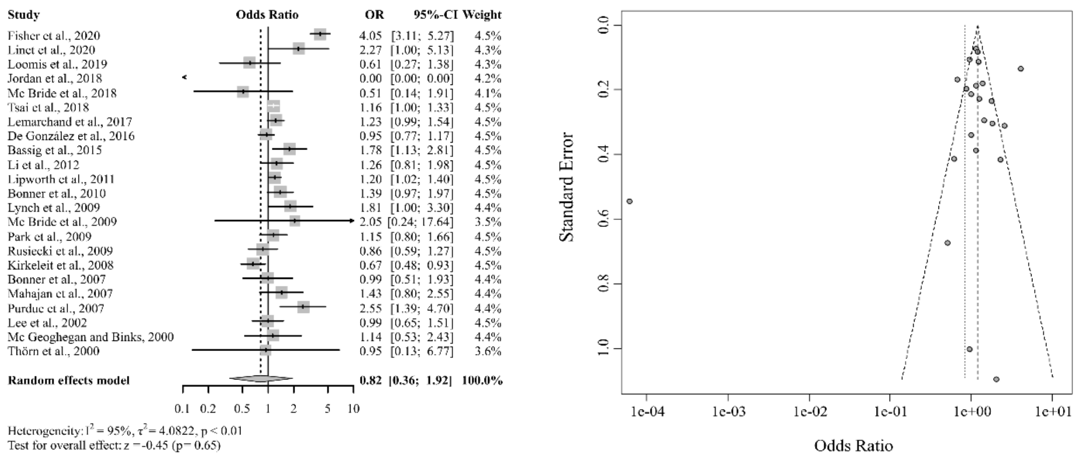


**Figure S4:** Forest Plot and Funnel Plot of the (A) Case-control [2,5,7,8,10–13,17,18,21,23,25–31,33–41]; and (B) Cohort and cross-sectional studies included in the meta-analysis [1,3,4,6,9,14–16,20,22,24,32,42–52].

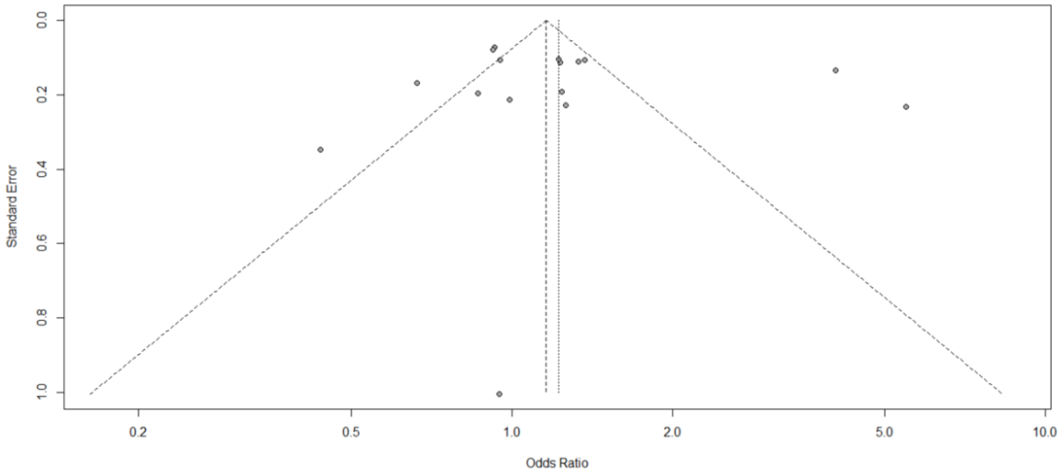


**Figure S5:** Funnel Plot of the studies included in the meta-analysis that evaluated only work class. Each point represents one study.

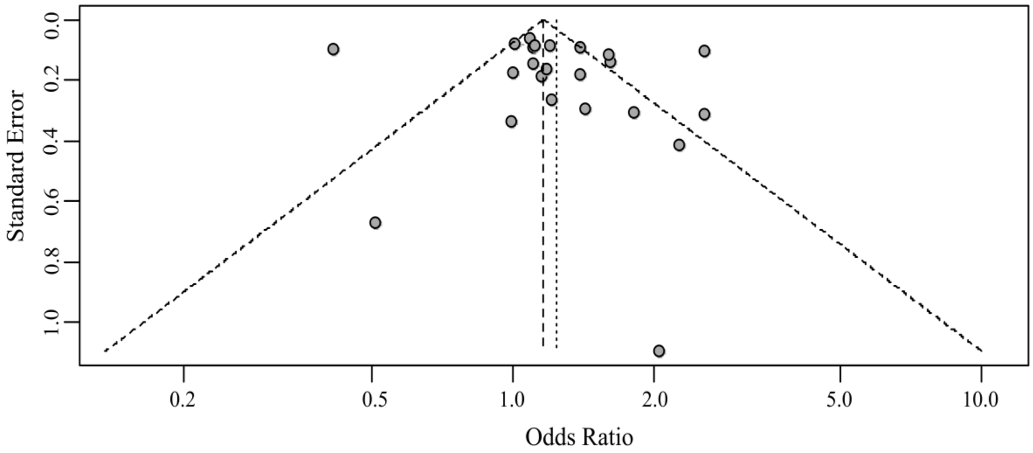


**Figure S6:** Funnel Plot of the studies included in the meta-analysis that evaluated only chemical compounds. Each point represents one study.

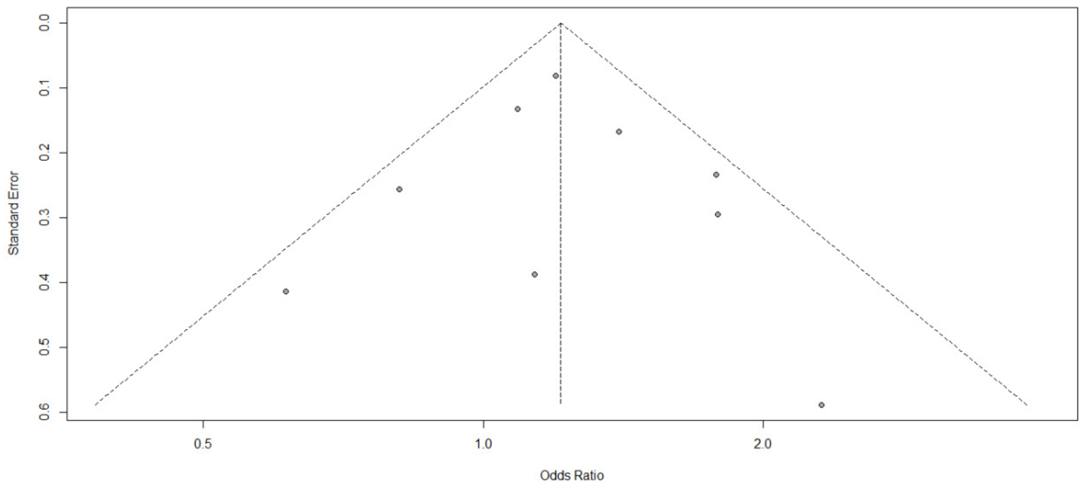


**Figure S7:** Funnel Plot of the studies included in the meta-analysis that simultaneously evaluated work class and exposure to chemical compounds. Each dot represents one study.

## References

1. Fisher, J.A.; Freeman, L.E.B.; Hofmann, J.N.; Blair, A.; Parks, C.G.; Thorne, P.S.; Ward, M.H.; Jones, R.R. Residential Proximity to Intensive Animal Agriculture and Risk of Lymphohematopoietic Cancers in the Agricultural Health Study. *Epidemiology* **2020**, *31*, 478–489, doi:10.1097/EDE.0000000000001186.
2. Satta, G.; Loi, M.; Becker, N.; Benavente, Y.; De Sanjose, S.; Foretova, L.; Staines, A.; Maynadie, M.; Nieters, A.; Meloni, F.; et al. Occupational Exposure to Ionizing Radiation and Risk of Lymphoma Subtypes: Results of the Epilymph European Case-Control Study. *Environ Health* **2020**, *19*, 43, doi:10.1186/s12940-020-00596-9.
3. Loomis, D.; Guha, N.; Kogevinas, M.; Fontana, V.; Gennaro, V.; Kolstad, H.A.; McElvenny, D.M.; Sallmén, M.; Saracci, R. Cancer Mortality in an International Cohort of Reinforced Plastics Workers Exposed to Styrene: A Reanalysis. *Occup Environ Med* **2019**, *76*, 157–162, doi:10.1136/oemed-2018-105131.
4. McBride, D.I.; Collins, J.J.; Bender, T.J.; Bodner, K.M.; Aylward, L.L. Cohort Study of Workers at a New Zealand Agrochemical Plant to Assess the Effect of Dioxin Exposure on Mortality. *BMJ Open* **2018**, *8*, e019243, doi:10.1136/bmjopen-2017-019243.
5. Ferri, G.M.; Specchia, G.; Mazza, P.; Ingravallo, G.; Intranuovo, G.; Guastadisegno, C.M.; Congedo, M.L.; Lagioia, G.; Loparco, M.C.; Giordano, A.; et al. Risk of Lymphoma Subtypes by Occupational Exposure in Southern Italy. *J Occup Med Toxicol* **2017**, *12*, doi:10.1186/s12995-017-0177-2.
6. González, A.B. de; Ntowe, E.; Kitahara, C.M.; Gilbert, E.; Miller, D.L.; Kleinerman, R.A.; Linet, M.S. Long-Term Mortality in 43 763 U.S. Radiologists Compared with 64 990 U.S. Psychiatrists. *Radiology* **2016**, *281*, 847, doi:10.1148/radiol.2016152472.
7. Kachuri, L.; Demers, P.A.; Blair, A.; Spinelli, J.J.; Pahwa, M.; McLaughlin, J.R.; Pahwa, P.; Dosman, J.A.; Harris, S.A. Multiple Pesticide Exposures and the Risk of Multiple Myeloma in Canadian Men. *International Journal of Cancer* **2013**, *133*, 1846–1858, doi:10.1002/ijc.28191.
8. Karunanayake, C.P.; Dosman, J.A.; Pahwa, P. Non-Hodgkin's Lymphoma and Work in Agriculture: Results of a Two Case-Control Studies in Saskatchewan, Canada. *Indian J Occup Environ Med* **2013**, *17*, 114–121, doi:10.4103/0019-5278.130860.

9. Li, J.; Cone, J.E.; Kahn, A.R.; Brackbill, R.M.; Farfel, M.R.; Greene, C.M.; Hadler, J.L.; Stayner, L.T.; Stellman, S.D. Association Between World Trade Center Exposure and Excess Cancer Risk. *JAMA* **2012**, *308*, 2479–2488, doi:10.1001/jama.2012.110980.
10. Zakerinia, M.; Namdari, M.; Amirghofran, S. The Relationship between Exposure to Pesticides and the Occurrence of Lymphoid Neoplasm. *Iran Red Crescent Med J* **2012**, *14*, 337–344.
11. Moore, T.; Brennan, P.; Becker, N.; de Sanjosé, S.; Maynadié, M.; Foretova, L.; Cocco, P.; Staines, A.; Nieters, A.; Font, R.; et al. Occupational Exposure to Meat and Risk of Lymphoma: A Multicenter Case-Control Study from Europe. *International Journal of Cancer* **2007**, *121*, 2761–2766, doi:10.1002/ijc.22994.
12. Cocco, P.; t'Mannetje, A.; Fadda, D.; Melis, M.; Becker, N.; de Sanjosé, S.; Foretova, L.; Mareckova, J.; Staines, A.; Kleefeld, S.; et al. Occupational Exposure to Solvents and Risk of Lymphoma Subtypes: Results from the Epilymph Case-Control Study. *Occup Environ Med* **2010**, *67*, 341–347, doi:10.1136/oem.2009.046839.
13. Seidler, A.; Becker, N.; Nieters, A.; Arhelger, R.; Mester, B.; Rossnagel, K.; Deeg, E.; Elsner, G.; Melis, M.; Sesler, S.; et al. Asbestos Exposure and Malignant Lymphoma: A Multicenter Case–Control Study in Germany and Italy. *Int Arch Occup Environ Health* **2010**, *83*, 563–570, doi:10.1007/s00420-009-0502-x.
14. McBride, D.I.; Collins, J.J.; Humphry, N.F.; Herbison, P.; Bodner, K.M.; Aylward, L.L.; Burns, C.J.; Wilken, M. Mortality in Workers Exposed to 2,3,7,8-Tetrachlorodibenzo-p-Dioxin at a Trichlorophenol Plant in New Zealand. *J Occup Environ Med* **2009**, *51*, 1049–1056, doi:10.1097/JOM.0b013e3181b571ae.
15. Rusiecki, J.A.; Patel, R.; Koutros, S.; Beane-Freeman, L.; Landgren, O.; Bonner, M.R.; Coble, J.; Lubin, J.; Blair, A.; Hoppin, J.A.; et al. Cancer Incidence among Pesticide Applicators Exposed to Permethrin in the Agricultural Health Study. *Environ Health Perspect* **2009**, *117*, 581–586, doi:10.1289/ehp.11318.
16. Kirkeleit, J.; Riise, T.; Bråtveit, M.; Moen, B.E. Increased Risk of Acute Myelogenous Leukemia and Multiple Myeloma in a Historical Cohort of Upstream Petroleum Workers Exposed to Crude Oil. *Cancer Causes Control* **2008**, *19*, 13–23, doi:10.1007/s10552-007-9065-x.
17. Balen, E. van; Font, R.; Cavallé, N.; Font, L.; Garcia-Villanueva, M.; Benavente, Y.; Brennan, P.; Sanjose, S. de Exposure to Non-Arsenic Pesticides Is Associated with Lymphoma among Farmers in Spain. *Occupational and Environmental Medicine* **2006**, *63*, 663–668, doi:10.1136/oem.2005.024026.
18. Fritschi, L.; Benke, G.; Hughes, A.M.; Kricker, A.; Turner, J.; Vajdic, C.M.; Grulich, A.; Milliken, S.; Kaldor, J.; Armstrong, B.K. Occupational Exposure to Pesticides and Risk of Non-Hodgkin's Lymphoma. *Am J Epidemiol* **2005**, *162*, 849–857, doi:10.1093/aje/kwi292.
19. Lee, E.; Burnett, C.A.; Lalich, N.; Cameron, L.L.; Sestito, J.P. Proportionate Mortality of Crop and Livestock Farmers in the United States, 1984–1993. *American Journal of Industrial Medicine* **2002**, *42*, 410–420, doi:10.1002/ajim.10131.
20. McGeoghegan, D.; Binks, K. The Mortality and Cancer Morbidity Experience of Workers at the Springfields Uranium Production Facility, 1946-95. *J Radiol Prot* **2000**, *20*, 111–137, doi:10.1088/0952-4746/20/2/301.
21. Benavente, Y.; Costas, L.; Rodríguez-Suarez, M.M.; Alguacil, J.; Santibáñez, M.; Vila, J.; Robles, C.; Alonso, E.; de la Banda, E.; Gonzalez-Barca, E.; et al. Occupational Exposure to Pesticides and Chronic Lymphocytic Leukaemia in the MCC-Spain Study. *Int J Environ Res Public Health* **2020**, *17*, 5174, doi:10.3390/ijerph17145174.
22. Lemarchand, C.; Tual, S.; Levêque-Morlais, N.; Perrier, S.; Belot, A.; Velten, M.; Guizard, A.-V.; Marcotullio, E.; Monnereau, A.; Clin, B.; et al. Cancer Incidence in the AGRICAN Cohort Study (2005-2011). *Cancer Epidemiol* **2017**, *49*, 175–185, doi:10.1016/j.canep.2017.06.003.

23. Cocco, P.; Vermeulen, R.; Flore, V.; Nonne, T.; Campagna, M.; Purdue, M.; Blair, A.; Monnereau, A.; Orsi, L.; Clavel, J.; et al. Occupational Exposure to Trichloroethylene and Risk of Non-Hodgkin Lymphoma and Its Major Subtypes: A Pooled IinterLlymph Analysis. *Occup Environ Med* **2013**, *70*, 795–802, doi:10.1136/oemed-2013-101551.
24. Lipworth, L.; Sonderman, J.S.; Mumma, M.T.; Tarone, R.E.; Marano, D.E.; Boice, J.D.J.; McLaughlin, J.K. Cancer Mortality Among Aircraft Manufacturing Workers: An Extended Follow-Up. *Journal of Occupational and Environmental Medicine* **2011**, *53*, 992–1007, doi:10.1097/JOM.0b013e31822e0940.
25. Purdue, M.P.; Bakke, B.; Stewart, P.; De Roos, A.J.; Schenk, M.; Lynch, C.F.; Bernstein, L.; Morton, L.M.; Cerhan, J.R.; Severson, R.K.; et al. A Case–Control Study of Occupational Exposure to Trichloroethylene and Non-Hodgkin Lymphoma. *Environ Health Perspect* **2011**, *119*, 232–238, doi:10.1289/ehp.1002106.
26. Kiran, S.; Cocco, P.; Mannetje, A.; Satta, G.; D'Andrea, I.; Becker, N.; de Sanjosé, S.; Foretova, L.; Staines, A.; Kleefeld, S.; et al. Occupational Exposure to Ethylene Oxide and Risk of Lymphoma. *Epidemiology* **2010**, *21*, 905–910, doi:10.1097/EDE.0b013e3181f4cc0f.
27. Wong, O.; Harris, F.; Wang, Y.; Fu, H. A Hospital-Based Case-Control Study of Non-Hodgkin Lymphoid Neoplasms in Shanghai: Analysis of Personal Characteristics, Lifestyle, and Environmental Risk Factors by Subtypes of the WHO Classification. *J Occup Environ Med* **2010**, *52*, 39–53, doi:10.1097/JOM.0b013e3181c5c399.
28. Wang, R.; Zhang, Y.; Lan, Q.; Holford, T.R.; Leaderer, B.; Hoar Zahm, S.; Boyle, P.; Dosemeci, M.; Rothman, N.; Zhu, Y.; et al. Occupational Exposure to Solvents and Risk of Non-Hodgkin Lymphoma in Connecticut Women. *Am J Epidemiol* **2009**, *169*, 176–185, doi:10.1093/aje/kwn300.
29. Tranah, G.J.; Bracci, P.M.; Holly, E.A. Domestic and Farm-Animal Exposures and Risk of Non-Hodgkin Lymphoma in a Population-Based Study in the San Francisco Bay Area. *Cancer Epidemiol Biomarkers Prev* **2008**, *17*, 2382–2387, doi:10.1158/1055-9965.EPI-08-0373.
30. Miligi, L.; Costantini, A.S.; Benvenuti, A.; Kriebel, D.; Bolejack, V.; Tumino, R.; Ramazzotti, V.; Rodella, S.; Stagnaro, E.; Crosignani, P.; et al. Occupational Exposure to Solvents and the Risk of Lymphomas. *Epidemiology* **2006**, *17*, 552–561, doi:10.1097/01.ede.0000231279.30988.4d.
31. Chiu, B.C.-H.; Weisenburger, D.D.; Zahm, S.H.; Cantor, K.P.; Gapstur, S.M.; Holmes, F.; Burmeister, L.F.; Blair, A. Agricultural Pesticide Use, Familial Cancer, and Risk of Non-Hodgkin Lymphoma. *Cancer Epidemiol Biomarkers Prev* **2004**, *13*, 525–531.
32. Lee, E.; Burnett, C.A.; Lalich, N.; Cameron, L.L.; Sestito, J.P. Proportionate Mortality of Crop and Livestock Farmers in the United States, 1984–1993. *American Journal of Industrial Medicine* **2002**, *42*, 410–420, doi:10.1002/ajim.10131.
33. Zheng, T.; Zahm, S.H.; Cantor, K.P.; Weisenburger, D.D.; Zhang, Y.; Blair, A. Agricultural Exposure to Carbamate Pesticides and Risk of Non-Hodgkin Lymphoma. *J Occup Environ Med* **2001**, *43*, 641–649, doi:10.1097/00043764-200107000-00012.
34. Boccolini, P. de M.M.; Boccolini, C.S.; Chrisman, J. de R.; Koifman, R.J.; Meyer, A. Non-Hodgkin Lymphoma among Brazilian Agricultural Workers: A Death Certificate Case-Control Study. *Archives of Environmental & Occupational Health* **2017**, *72*, 139–144, doi:10.1080/19338244.2016.1179167.
35. Xu, J.-X.; Hoshida, Y.; Yang, W.-I.; Inohara, H.; Kubo, T.; Kim, G.-E.; Yoon, J.-H.; Kojya, S.; Bandoh, N.; Harabuchi, Y.; et al. Life-Style and Environmental Factors in the Development of Nasal NK/T-Cell Lymphoma: A Case–Control Study in East Asia. *International Journal of Cancer* **2007**, *120*, 406–410, doi:10.1002/ijc.22313.
36. Kato, I.; Koenig, K.L.; Watanabe-Meserve, H.; Baptiste, M.S.; Lillquist, P.P.; Frizzera, G.; Burke, J.S.; Moseson, M.; Shore, R.E. Personal and Occupational Exposure to Organic Solvents and Risk of Non-Hodgkin's Lymphoma (NHL) in Women (United States). *Cancer Causes Control* **2005**, *16*, 1215–1224, doi:10.1007/s10552-005-0385-4.

37. Kato, I.; Watanabe-Meserve, H.; Koenig, K.L.; Baptiste, M.S.; Lillquist, P.P.; Frizzera, G.; Burke, J.S.; Moseson, M.; Shore, R.E. Pesticide Product Use and Risk of Non-Hodgkin Lymphoma in Women. *Environ Health Perspect* **2004**, *112*, 1275–1281, doi:10.1289/ehp.7070.
38. De Roos, A.J.; Zahm, S.; Cantor, K.; Weisenburger, D.; Holmes, F.; Burmeister, L.; Blair, A. Integrative Assessment of Multiple Pesticides as Risk Factors for Non-Hodgkin's Lymphoma among Men. *Occup Environ Med* **2003**, *60*, e11, doi:10.1136/oem.60.9.e11.
39. Fritschi, L.; Johnson, K.C.; Kliewer, E.V.; Fry, R.; Group, C.C.R.E.R. Animal-Related Occupations and the Risk of Leukemia, Myeloma, and Non-Hodgkin's Lymphoma in Canada. *Cancer Causes & Control* **2002**, *13*, 563–571.
40. McDuffie, H.H.; Pahwa, P.; McLaughlin, J.R.; Spinelli, J.J.; Fincham, S.; Dosman, J.A.; Robson, D.; Skinnider, L.F.; Choi, N.W. Non-Hodgkin's Lymphoma and Specific Pesticide Exposures in Men: Cross-Canada Study of Pesticides and Health. *Cancer Epidemiol Biomarkers Prev* **2001**, *10*, 1155–1163.
41. van Wijngaarden, E.; Savitz, D.A. Occupational Sunlight Exposure and Mortality from Non-Hodgkin Lymphoma among Electric Utility Workers. *J Occup Environ Med* **2001**, *43*, 548–553, doi:10.1097/00043764-200106000-00008.
42. Linet, M.S.; Gilbert, E.S.; Vermeulen, R.; Dores, G.M.; Yin, S.-N.; Portengen, L.; Hayes, R.B.; Ji, B.-T.; Lan, Q.; Li, G.-L.; et al. Benzene Exposure-Response and Risk of Lymphoid Neoplasms in Chinese Workers: A Multicenter Case-Cohort Study. *American Journal of Industrial Medicine* **2020**, *63*, 741–754, doi:10.1002/ajim.23142.
43. Jordan, H.T.; Stein, C.R.; Li, J.; Cone, J.E.; Stayner, L.; Hadler, J.L.; Brackbill, R.M.; Farfel, M.R. Mortality among Rescue and Recovery Workers and Community Members Exposed to the September 11, 2001 World Trade Center Terrorist Attacks, 2003–2014. *Environmental Research* **2018**, *163*, 270–279, doi:10.1016/j.envres.2018.01.004.
44. Tsai, S.-S.; Weng, Y.-H.; Chiu, Y.-W.; Yang, C.-Y. Farming and Mortality Rates Attributed to Non-Hodgkin's Lymphoma in Taiwan. *J. Toxicol. Environ. Health Part A* **2018**, *81*, 31–36, doi:10.1080/15287394.2017.1408362.
45. Bassig, B.A.; Friesen, M.C.; Vermeulen, R.; Shu, X.-O.; Purdue, M.P.; Stewart, P.A.; Xiang, Y.-B.; Chow, W.-H.; Zheng, T.; Ji, B.-T.; et al. Occupational Exposure to Benzene and Non-Hodgkin Lymphoma in a Population-Based Cohort: The Shanghai Women's Health Study. *Environ Health Perspect* **2015**, *123*, 971–977, doi:10.1289/ehp.1408307.
46. Bonner, M.R.; Williams, B.A.; Rusiecki, J.A.; Blair, A.; Freeman, L.E.B.; Hoppin, J.A.; Dosemeci, M.; Lubin, J.; Sandler, D.P.; Alavanja, M.C.R. Occupational Exposure to Terbufos and the Incidence of Cancer in the Agricultural Health Study. *Cancer Causes Control* **2010**, *21*, 871–877, doi:10.1007/s10552-010-9514-9.
47. Lynch, S.M.; Mahajan, R.; Freeman, L.E.B.; Hoppin, J.A.; Alavanja, M.C.R. Cancer Incidence among Pesticide Applicators Exposed to Butylate in the Agricultural Health Study (AHS). *Environ Res* **2009**, *109*, 860–868, doi:10.1016/j.envres.2009.06.006.
48. Park, S.K.; Kang, D.; Beane-Freeman, L.; Gwak, J.; Hoppin, J.A.; Sandler, D.P.; Knott, C.; Lynch, C.F.; Blair, A.; Alavanja, M. Cancer Incidence among Paraquat-Exposed Pesticide Applicators in the Agricultural Health Study. *Int J Occup Environ Health* **2009**, *15*, 274–281.
49. Bonner, M.R.; Coble, J.; Blair, A.; Beane Freeman, L.E.; Hoppin, J.A.; Sandler, D.P.; Alavanja, M.C.R. Malathion Exposure and the Incidence of Cancer in the Agricultural Health Study. *Am J Epidemiol* **2007**, *166*, 1023–1034, doi:10.1093/aje/kwm182.
50. Mahajan, R.; Blair, A.; Coble, J.; Lynch, C.F.; Hoppin, J.A.; Sandler, D.P.; Alavanja, M.C.R. Carbaryl Exposure and Incident Cancer in the Agricultural Health Study. *Int J Cancer* **2007**, *121*, 1799–1805, doi:10.1002/ijc.22836.
51. Purdue, M.P.; Hoppin, J.A.; Blair, A.; Dosemeci, M.; Alavanja, M.C.R. Occupational Exposure to Organochlorine Insecticides and Cancer Incidence in the Agricultural Health Study. *Int J Cancer* **2007**, *120*, 642–649, doi:10.1002/ijc.22258.

52. Thörn, Å.; Gustavsson, P.; Sadigh, J.; Westerlund-Hännestrand, B.; Hogstedt, C. Mortality and Cancer Incidence among Swedish Lumberjacks Exposed to Phenoxy Herbicides. *Occupational and Environmental Medicine* **2000**, *57*, 718–720, doi:10.1136/oem.57.10.718.
